# Transmission Potential of Rift Valley Fever Virus over the Course of the 2010 Epidemic in South Africa

**DOI:** 10.3201/eid1906.121641

**Published:** 2013-06

**Authors:** Raphaëlle Métras, Marc Baguelin, W. John Edmunds, Peter N. Thompson, Alan Kemp, Dirk U. Pfeiffer, Lisa M. Collins, Richard G. White

**Affiliations:** Royal Veterinary College, Hatfield, UK (R. Métras, D.U. Pfeiffer);; London School of Hygiene and Tropical Medicine, London, UK (R. Métras, M. Baguelin, W.J. Edmunds, R.G. White);; Health Protection Agency, London (M. Baguelin);; University of Pretoria, Pretoria, South Africa (P.N. Thompson);; National Institute for Communicable Diseases, Sandringham, South Africa (A. Kemp);; Queen’s University Belfast, Belfast, Northern Ireland, UK (L.M. Collins)

**Keywords:** Rift Valley fever, South Africa, epidemic, likelihood functions, viruses, Rift Valley fever virus, zoonoses, transmission

## Abstract

A Rift Valley fever (RVF) epidemic affecting animals on domestic livestock farms was reported in South Africa during January–August 2010. The first cases occurred after heavy rainfall, and the virus subsequently spread countrywide. To determine the possible effect of environmental conditions and vaccination on RVF virus transmissibility, we estimated the effective reproduction number (*R_e_*) for the virus over the course of the epidemic by extending the Wallinga and Teunis algorithm with spatial information. *R_e_* reached its highest value in mid-February and fell below unity around mid-March, when vaccination coverage was 7.5%–45.7% and vector-suitable environmental conditions were maintained. The epidemic fade-out likely resulted first from the immunization of animals following natural infection or vaccination. The decline in vector-suitable environmental conditions from April onwards and further vaccination helped maintain *R_e_* below unity. Increased availability of vaccine use data would enable evaluation of the effect of RVF vaccination campaigns.

Rift Valley fever (RVF) is a zoonotic arbovirosis caused by infection with a phlebovirus (family *Bunyaviridae*, genus *Phlebovirus*). The main vectors are specific *Aedes* and *Culex* spp. mosquitoes, and primary hosts are sheep, goats, and cattle (*1*,*2*). RVF epidemics usually occur after heavy rainfalls, which inundate ephemeral wetlands and enable large numbers of *Aedes* spp. mosquito eggs to hatch; it has been hypothesized that these mosquitoes harbor RFV virus (*3*–*5*). Virus transmission is sustained in locations with more persistent surface water, which provides suitable breeding conditions for other vectors, such as *Culex* sp. mosquitoes (*6*). RVF epidemics among animal herds cause abortion storms, affecting all stages of pregnancy, and high death rates among neonates. Epidemics among humans often cause influenza-like illness, although severe conditions (e.g., hemorrhagic fever and death) have been reported (*1*,*2*).

RVF epidemics occurred in South Africa in 1950–1951 (*7*), 1973–1975 (*8*), and 2010–2011. The 2010 wave started in January and February in Free State Province and subsequently spread to almost all provinces in South Africa (Figure 1, panel A). Animals from a variety of species were affected (e.g., cattle, sheep and goats, buffaloes, camels, and other wild animals), and 95% (n = 470) of the affected farms raised cattle, small ruminants (sheep/goats), or both (*9*). The incidence peaked in March, and the last case of that wave was reported in August 2010. The epidemic resumed in January 2011, affecting 124 farms, mainly in Eastern Cape Province (Figure 1, panel B) (*10*). The start of the 2010 epidemic was attributed to heavy rainfall in January and February (*11*,*12*). The fade-out of the 2010 wave could be attributed to several factors: a depletion of susceptible hosts after natural infection or vaccination (*13*); a change of environmental conditions affecting the sustainability of vector breeding, such as a decrease in temperature (*14*); the drying of wetlands; or a combination of these factors.

**Figure 1:**
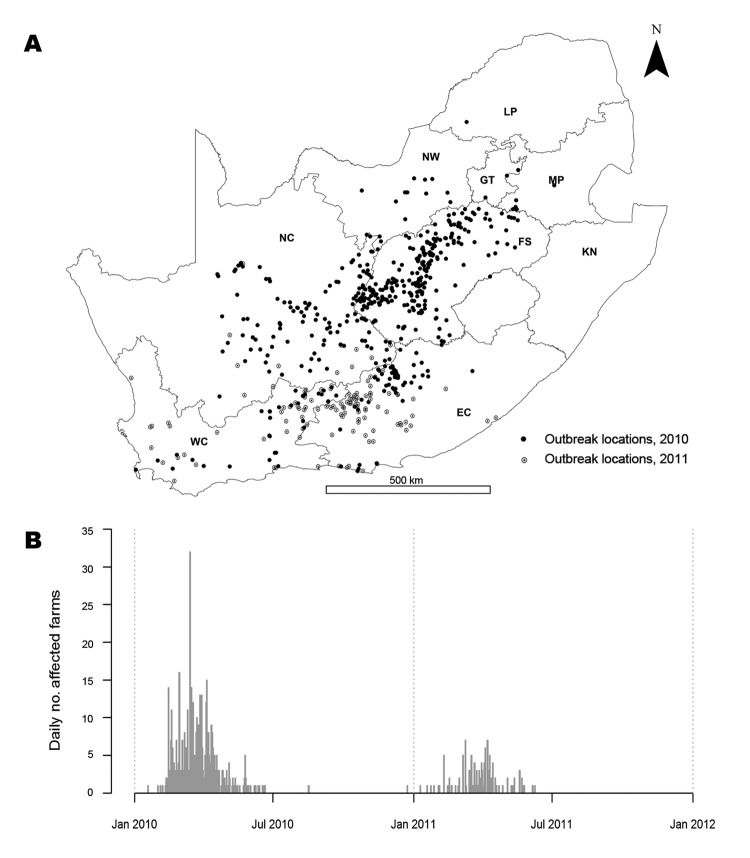
Rift Valley fever epidemic, South Africa, 2010–2011. A) Location of cases. Unmarked area in center right is Lesotho (no data). B) Epidemic curve for the 2 years. NC, Northern Cape; NW, North West; LP, Limpopo; GT, Gauteng; MP, Mpumalanga; FS, Free State; KN, KwaZulu-Natal; EC, Eastern Cape; WC, Western Cape.

The effective reproduction number (*R_e_*) is a key epidemiologic parameter that measures the transmission potential of the causative agent of a disease during an epidemic. *R_e_* is defined by the number of secondary infections resulting from 1 infectious case in a population in which some members are already immune (*15*). When *R_e_* is above 1, the infection spreads; maintenance of *R_e_* below 1 is required to stop an outbreak (*16*).

The objective of this study was to estimate *R_e_* at the farm level over the course of the 2010 RVF epidemic wave in South Africa by applying the Wallinga and Teunis transmission tree–reconstruction method (*17*), extended to use geographic information. By tracking the transmission potential of the virus and comparing our findings with data on vaccination and climate (rainfall and temperature), we determined plausible reasons for fade-out of the epidemic wave.

## Methods

### RVF Dataset and Study Period

A total of 470 RVF cases were reported over the study period (January–August 2010). A case was defined as an outbreak reported from a farm raising cattle, small ruminants, or both (*9*). Available information comprised the global positioning system coordinates and outbreak starting dates for the affected farms.

### Estimation of Effective Reproduction Number

**Figure 2:**
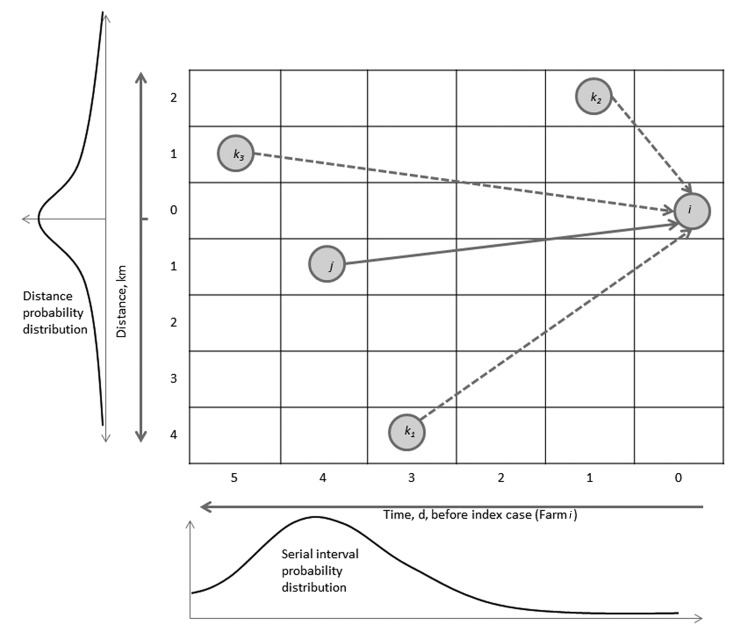
Schematic representation of the Wallinga-Teunis algorithm extended with spatial information. Farm *i* could get infection from Farm *j*, but it also could get infection from Farms *k_1_*, *k_2_*, and *k_3_*. In this example, the most likely time difference between onset of symptoms is 4 days (based on the serial interval distribution, given below the *x*-axis), and the most likely distance between farms is short (<1 km). Therefore, Farm *j* is the most likely farm to have infected Farm *i* (this scenario maximizes the probability in both dimensions). See the online Technical Appendix (wwwnc.cdc.gov/EID/article/19/6/12-1641-Techapp1.pdf) for details.

The Wallinga and Teunis method (*17*), extended with spatial information, enables estimation of *R_e_* at the farm level by calculating the relative likelihood, or probability (*p_ij_*), that a specific farm (*i*) gets infected from another specific farm (*j*). This probability, *p_ij_*, is equal to the probability that farm *j* infects farm *i*, divided by the probability that farm *i* had been infected from any other farm (*k*) in the dataset (Figure 2). These probabilities depend on the number of days separating the onset of symptoms on the 2 farms (*i* and *j*) and the distance (in kilometers) separating *i* and *j*, and the probabilities were extracted from a probability density function of the generation interval (Technical Appendix). The generation (or serial) interval was defined as the time between onset of symptoms for a primary case and the onset of symptoms for its secondary case (*18*). In the stylized example in Figure 2, the most likely time difference was 4 days (determined on the basis of the serial interval distribution, given below the *x* axis), and the most likely distance is short (<1 km). Therefore, farm *j* is the most likely farm to have infected farm *i* (this maximized the probability in both dimensions).

Because no independent dataset (i.e., from another epidemic in another country) was available to estimate a generation interval for RVF at the farm level and in 2 dimensions (i.e., distance and time), we used the dataset for the 2011 RVF outbreak in South Africa. In a previous analysis, Métras et al. (*19*) estimated the spatiotemporal interaction (or proximity) from the 2011 dataset [denoted *D_0_*(*s,t*)] by using the space–time *K*-function (*20*). These *D_0_*(*s,t*) values were used as a generation interval distribution to calculate *p_ij_* (online Technical Appendix).

### Sensitivity Analysis

**Figure 3:**
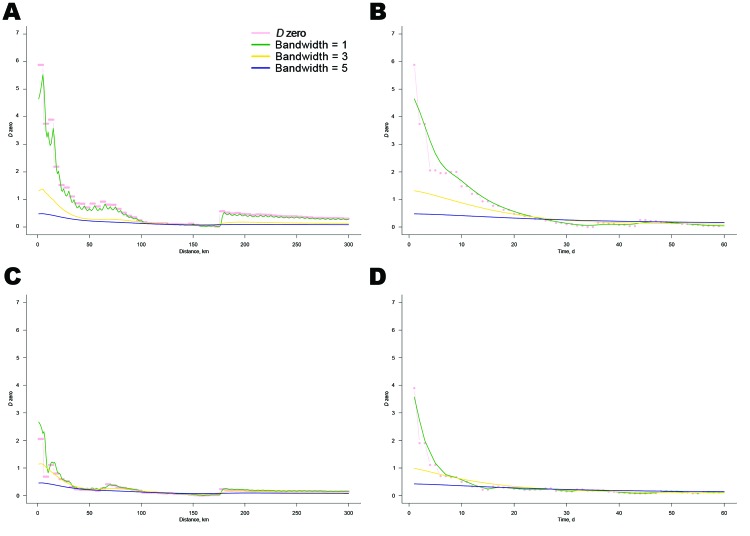
Distribution of *D*_0_ by time and distance [*D_0_*(*s,t*)]. *D_0_*(*s,t*) is a measure of spatiotemporal interaction between cases that was estimated by using the space–time *K*-function (*19*,*20*); the distribution is indicated by the pink dashed line. The green, yellow, and blue lines are the smoothed distributions, which were obtained with bandwidth values of 1, 3, and 5, respectively. A) Plot of *D_0_*(*s,t*) values by distance on day 1. B) *D_0_*(*s,t*) values by time at distance of 5 km. C) Plot of *D_0_*(*s,t*) values by distance on day 5. D) *D_0_*(*s,t*) values by time at distance of 15 km.

The shape of the *D_0_*(*s,t*) plot, peaking for short space–time windows (Figure 3), suggested that most of the transmission was attributed to short-distance mechanisms (e.g., local vector dispersal) rather than long-distance mechanisms (e.g., movement of infectious animals or wind carriage of vectors) (*19*). By using this generation interval for the duration of the epidemic, a constant and high importance of short-distance transmission mechanisms was assumed. However, as the epidemic grew, these short-distance transmission mechanisms were likely to be less important; or in, other words, as farms around a case became infected and immune, short-distance transmission was likely to be less involved in disease spread. Thus, we investigated the variations of *R_e_* by giving less weight to short-distance transmission and more weight to long-distance transmission. To obtain such serial interval distributions, the *D_0_*(*s,t*) distribution was flattened by using a 2-dimensional double exponential kernel function with bandwidth values equal to 1, 3, and 5, resulting in 3 smoothed surfaces (Figure 3). It was assumed that the bandwidth equal to 1 would better correspond to the serial interval distribution at the early stage of the epidemic and that bandwidth values 3 and 5 would better describe the intensity of the transmission when the population started to be immune (i.e., at the later stages of the epidemic).

### Vaccination Coverage and Climate Data

We collected information on animal vaccination and climate to determine the potential effect of these factors on the fade-out of the 2010 RVF epidemic. RVF vaccination in South Africa is not compulsory and is not implemented by the government. Although the government can strongly advise farmers to vaccinate their animals, implementation of vaccination on a farm depends on the individual farmer’s decision. Therefore, data on vaccination are especially limited.

Onderstepoort Biologic Products Ltd. (Onderstepoort, South Africa), the sole provider of RVF vaccine in South Africa, calculates its yearly sales from April of one year to March of the next year (*21*). During April 1, 2009–March 31, 2010, ≈3.4 million RVF vaccine doses (live attenuated Smithburn and inactivated) were sold, and during April 1–May 31, 2010, ≈5.8 million doses were sold (Table 1) (*22*). In our study, Period 1 corresponded with the time before the 2010 epidemic (April 1, 2009–January 18, 2010); Period 2 corresponded with the start of the 2010 epidemic and the end of the 2009 vaccine sales year (January 19, 2010–March 31, 2010); and Period 3 corresponded with April 1, 2010–May 31, 2010, beyond which no vaccine sales data were available (Table 1). Vaccination coverage was estimated up to March 31, 2010 (end of Period 2) and up to May 31, 2010 (end of Period 3). Since no spatial (i.e., location-specific) information on vaccine sales was available, vaccination coverage was estimated under 3 scenarios (A, B, and C): Scenario A assumed that vaccination coverage was applied throughout South Africa proportional to the livestock population; Scenario B assumed that the number of vaccines used in a province over a specific period was proportional to the number of cases reported in that province over that same period; Scenario C assumed that all vaccines were used in Free State Province during Periods 2 and 3 and that no vaccine had been used before the epidemic (Period 1). Therefore, using Scenario C, we could estimate the maximum coverage for Free State Province, which was the first and most affected province and also the one in which the government strongly supported vaccination (*13*). Formulas used to calculate vaccination coverage are available in the online Technical Appendix.

**Table 1:** Number of farms affected by Rift Valley fever before and during first 4.5 months of the 2010 epidemic, South Africa

Province	No. (%) farms affected				
	Before the epidemic		First 4.5 months of the epidemic		
	Period 1, April 1, 2009–January 18, 2010*		Period 2, January 19–March 31, 2010*	Period 3, April 1–May 31, 2010†	Periods 2 and 3, January 19–May 31, 2010
Free State	0 (0)		208 (66.9)	41 (27.2)	249 (53.9)
Northern Cape	19 (67.9)		61 (19.6)	54 (35.8)	115 (24.9)
Eastern Cape	0		24 (7.7)	26 (17.2)	50 (10.9)
Kwazulu-Natal	8 (28.6)		0	0 (0)	0
North West	0		7 (2.3)	8 (5.3)	15 (3.2)
Mpumalanga	1 (3.6)		5 (1.6)	0	5 (1.1)
Western Cape	0		4 (1.3)	20 (13.2)	24 (5.2)
Gauteng	0		2 (0.6)	1 (0.7)	3 (0.6)
Limpopo	0		0	1 (0.7)	1 (0.2)
All provinces	28 (100.0)		311 (100.0)	151 (100.0)	462 (100.0)

*A total of 3.4 million Rift Valley fever vaccine doses were sold during Periods 1 and 2. †5.8 million Rift Valley fever vaccine doses were sold during Period 3.

Most RVF cases were reported in Free State Province, although Northern Cape Province had the most cases in Period 3 (Table 1). Therefore, we averaged the daily minimum and maximum temperatures and total monthly rainfall from 5 weather stations in Free State (Bloemfontein, Kroonstad, Welkom, Fauresmith, and Gariep Dam) and 4 weather stations in Northern Cape (Kimberley, Prieska, De Aar, and Noupoort) (South African Weather Service, pers. comm.).

### Herd Immunity Threshold

Herd immunity threshold (HIT) is defined as the proportion of animals that needs to be immune to a pathogen to control transmission (*15*): 




In the equation, R_0_, the basic reproduction number, is the expected number of secondary cases generated by a primary case in a totally susceptible population and measures the potential for an infectious agent to start an outbreak. To compare the estimated vaccination coverage at the end of March with the proportion of farms on which animals should have been immune (either by natural infection or vaccination) to control transmission, we approximated HIT by replacing R_0_ in the equation by the highest value of *R_e_* (and its 95% CI values) at the start of the epidemic.

### Software

The analysis and plots were done by using R version 2.14.0 (*23*). Kernel smoothing was performed by using the image.smooth function in the fields package (*24*).

## Results

### Estimation of Effective Reproduction Number

**Figure 4:**
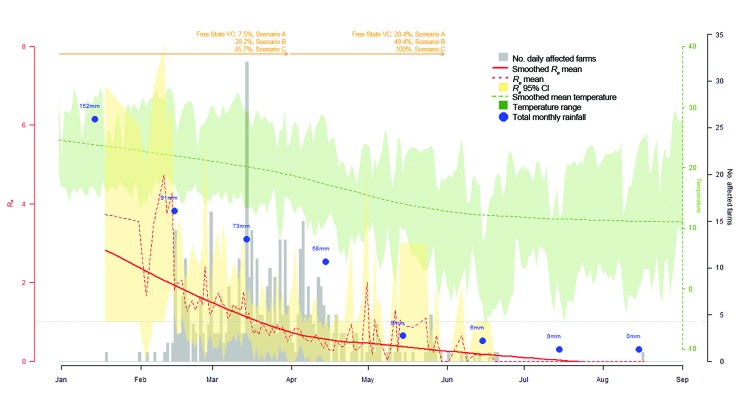
Rift Valley fever incidence (bars), daily effective reproduction number (*R_e;_* red dashed line), and smoothed mean of *R_e_* (solid red line) over the course of 2010 epidemic in Free State Province, South Africa. Blue dots, estimates of concurrent total monthly rainfall; dashed green line, average daily temperature. Vaccination coverage (VC) by March 31, 2010, and May 31, 2010, for Scenarios A–C (descriptions follow) are indicated at the top of the graph. Scenarios: Scenario A assumed that vaccination coverage was applied throughout South Africa in proportion to the livestock population; Scenario B assumed that the number of vaccines used in a province over a specific period was proportional to the number of cases reported in that province over that same period; Scenario C assumed that all vaccines were used in Free State Province during Period 2 (January 19–March 31, 2010) and Period 3 (April 1–May 31, 2010) and that no vaccine had been used before the epidemic (Period 1, April 1, 2009–January 18, 2010). The horizontal dashed line represents the threshold value *R_e_* = 1.

**Figure 5:**
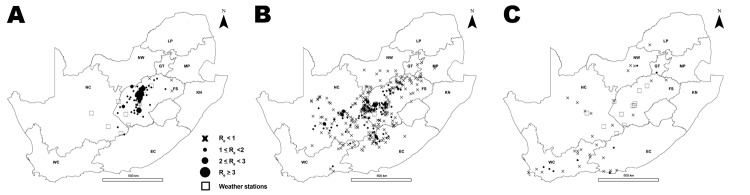
Effective reproduction number (*R_e_*) per affected farm, by province, over the 2010 Rift Valley fever epidemic, South Africa. A) January and February. B) March and April. C) May and June. July and August are not displayed because no cases were reported in July, and *R_e_* was 0 for the only farm reported in August. NC, Northern Cape; NW, North West; LP, Limpopo; GT, Gauteng; MP, Mpumalanga; FS, Free State; KN, KwaZulu-Natal; EC, Eastern Cape; WC, Western Cape. The unmarked area to the right of center is Lesotho (no data).

The estimated transmission potential of RVF virus from farms with infected animals peaked in mid-February (*R_e_* = 4.3, 95% CI 2.0–6.5), dropped sharply within a few days (*R_e_* = 1.8, 95% CI 1.21–2.43), and then remained at ≈1.5 until mid-March, at which time it dropped below unity, where it remained until the end of the epidemic (Figure 4). In addition, the lower bound of the 95% CI dropped and remained below 1.0 from mid-February onwards. In January and February, the most highly infectious farms (R_e_>2) were located in Free State Province (Figure 5, panels A–C), and although the data suggest the epidemic was still contained in Free State Province in February, a rapid fall in the *R_e_* value was observed (Figure 4). In March, the epidemic had spread to other provinces, mainly Northern Cape, and transmission was ongoing. In April, the spatial extent of the virus was similar to that in March, but most of the affected farms were not sources of ongoing transmission (*R_e_*<1). By May, only 7 spatially isolated farms had *R_e_* above unity.

**Figure 6:**
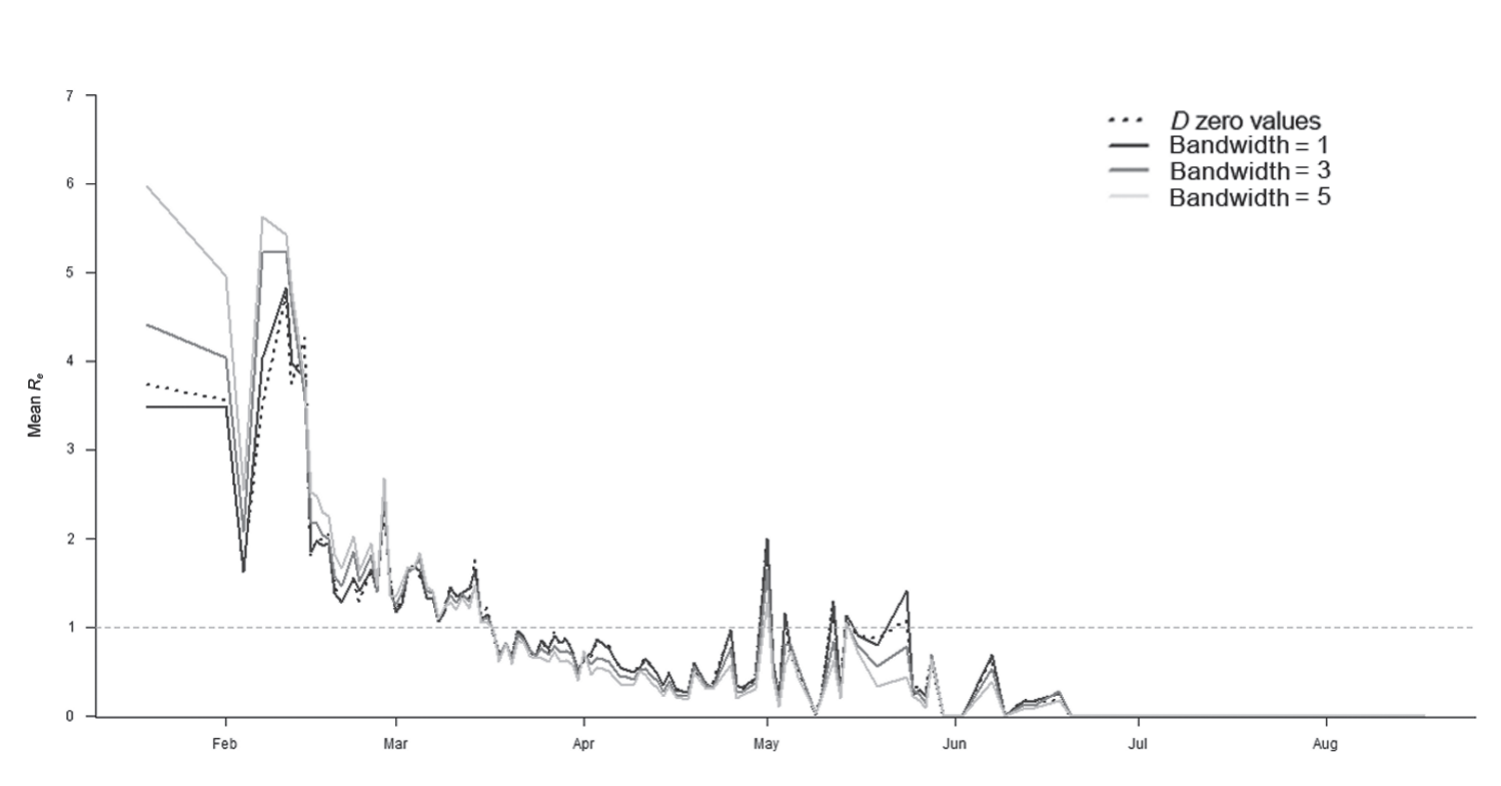
Mean effective daily reproduction number (*R_e_*) during Rift Valley fever epidemic, South Africa, 2010. *R_e_* was estimated by using *D_0_*(*s,t*) values (dashed black line) and *D_0_*(*s,t*) smoothed surfaces obtained with bandwidth values of 1 (dark gray), 3 (medium gray), and 5 (light gray). *D_0_*(*s,t*) values were estimated by using the space–time *K*-function (*19*,*20*) and are a measure of the spatiotemporal proximity between cases. The horizontal dashed line represents the threshold value *R_e_* = 1.

Figure 6 shows the variability of *R_e_* for the different serial interval distributions used in our analyses. In the early stages of the epidemic, *R_e_* was smaller when using input distributions that gave more weight to short-distance transmission [*D_0_*(*s,t*) and bandwidth 1] because it used only those cases closer in time and space, whereas when *R_e_* was estimated with flatter distributions (bandwidths 3 and 5), it also encompassed longer-distance transmission. However, for all distributions, the important variations of *R_e_* followed the same trend over time: a marked peak in January and February and stable transmission between late February and early March.

### Vaccine Coverage and Climate Data

At the end of March, we estimated vaccination coverage in Free State and Northern Cape Provinces to be 7.5% by applying vaccine coverage throughout the country in proportion to the livestock population (Scenario A, Table 2). When the number of vaccines used in each province was proportional to the number of RVF cases in each province, vaccination coverage was 28.2% in Free State and 11.0% in Northern Cape (Scenario B, Table 2). When all vaccines were used at the early stages of the epidemic in Free State Province only, vaccination coverage reached its highest value (45.7%) (Scenario C, Table 2). At the end of May, vaccination coverage in Free State was 20.4%, 49.4%, and 100.0% for Scenarios A, B, and C, respectively; vaccination coverage in Northern Cape Province was 39.6% for Scenario B (Table 2). In Scenario C, the total number of vaccines sold at the end of March was greater than the number of livestock in Free State Province. Thus, assuming that the spillover vaccine was used in Northern Cape, the estimated vaccination coverage in that province was 24.3%.

**Table 2:** Estimated vaccination coverage, under 3 different scenarios, during an epidemic of Rift Valley fever in 2 provinces in South Africa, 2010*

Scenario	% Vaccine coverage				
	Free State Province			Northern Cape Province	
	March 31	May 31		March 31	May 31
A	7.5	20.4		7.5	20.4
B	28.2	49.4		11.0	39.6
C	45.7	>100.0		0	0† (24.3‡)

*Scenario A assumed that vaccination coverage was applied throughout South Africa in proportion to the livestock population; Scenario B assumed that the number of vaccines used in a province over a specific period was proportional to the number of cases reported in that province over that same period; Scenario C assumed that all vaccines were used in Free State Province during Periods 2 (January 19–March 31, 2010) and 3 (April 1–May 31, 2010) and that no vaccine had been used before the epidemic (Period 1, April 1, 2009–January 18, 2010).           †Assumes that all vaccines are used in Free State. ‡Assumes that spillover vaccines from Free State were used in Northern Cape.

**Figure 7:**
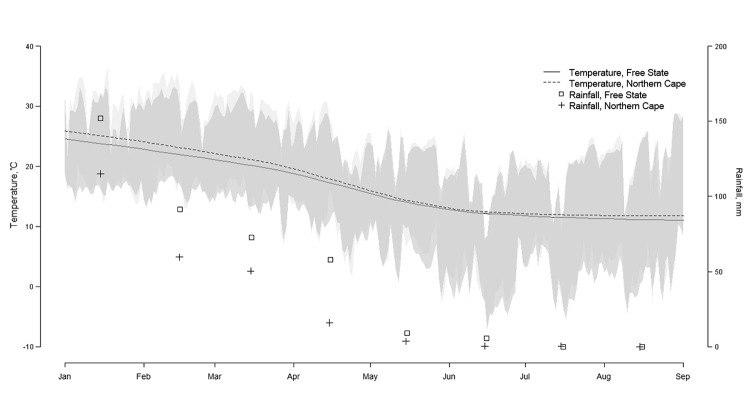
Daily temperature and total monthly rainfall during January–August 2010, Free State and Northern Cape Provinces, South Africa.

In Free State Province, monthly rainfall peaked in January (152 mm total). Substantial rainfall, although declining, persisted until April (58 mm total) and dropped in May (9 mm total), eventually approaching zero in September (Figure 4). The average daily temperature dropped from 24°C to 18°C during the study period; a decrease of 6°C (from 21°C to 15°C) occurred from mid-March to mid-May. Minimum daily temperatures fell below 13°C from early April onwards, but most of the time, the maximum daily temperature remained above 15°C. Rainfall and temperature estimates followed a similar trend in Free State and Northern Cape Provinces (Figure 7).

### Herd Immunity Threshold

In early February, the highest *R_e_* value was 4.3 (95% CI 2.0–6.5). The HIT at that time was therefore estimated at ≈78.9%, varying between 50.0% and 84.6%.

## Discussion

R_e_ reached its highest value in early February (*R_e_* = 4.3, 95% CI 2.0–6.5). Although *R_e_* fell below unity in mid-March, the lower bound of its 95% CI dropped below 1.0 in mid-February. Until the end of March, most RVF cases were recorded in Free State Province, and vaccination coverage was estimated between 7.5% and 45.7%. During this time, rainfall was substantial (73 mm total in March), so water was maintained in water bodies, and average temperature ranges (17°–24°C) were recorded (*14*). In addition, the minimum HIT was estimated at 50%. In April and May, *R_e_* was maintained below 1.0, more RVF cases were reported in Northern Cape, and vaccination coverage in Free State and Northern Cape varied between 20.4% and 100.0%. The level of rainfall was maintained until the end of April (58 mm total) and dropped to 9 mm in May; temperatures averaged below 20°C most days.

The *R_e_* peak observed in February followed the heavy rain observed in January, which, together with warm temperatures, created suitable environmental conditions for a massive hatching of *Aedes* spp. mosquito eggs (specifically, *Aedes juppi*, *Ae. caballus*, and *Ae. linneatopennis* in South Africa [*25*]) and initiation of the RVF outbreak. The virus originated from infected *Aedes* mosquito eggs (*5*,*26*) or possibly from other sources (e.g., long-distance vectors or movement of infected animals). Despite the decline in rainfall during January–March (from 152 mm to 73 mm/month) in Free State Province, transmission of RVF virus continued, although at a lower intensity. The continued transmission suggested that the lower amount of rainfall was sufficient to keep water bodies with good retention capacity filled and, thus, enable secondary vectors (e.g., *Cx. theileri* and *Anopheles cinereus*) (*25*) to sustain virus transmission in Free State and Northern Cape Provinces. From April onwards, the drop in rainfall may have contributed to a decreased abundance of *Culex* spp. mosquitoes; lower temperatures may have also slowed virus replication and shedding in *Culex* spp. vectors, as has been observed for *Cx. pipiens* (*27*,*28*), and thereby reduced virus transmission.

Given the environmental conditions, the RVF epidemic could have continued at least until the end of March in Free State Province. However, a depletion of susceptible animals after natural infection or vaccination probably caused the *R_e_* to fall below unity 2 weeks earlier (mid-March) and the lower bound of its 95% CI to fall in mid-February. In addition, the minimum HIT was estimated at 50.0%, but at the end of March, the estimated maximum vaccination coverage in Free State was 45.7% (Scenario C). By the end of May, vaccination coverage was higher, rainfall was very low, and temperatures continued to decrease, all of which probably contributed to preventing further virus transmission.

Several limitations with regard to the methods and data used might have altered the results of this study and their interpretation. First, the validity of the Wallinga and Teunis method assumes that all cases are reported and reported in a timely manner. The RVF cases used were those reported to the World Organisation for Animal Health. RVF is a notifiable disease that causes obvious signs in affected herds, so it is unlikely that underreporting was a major limitation. However, underreporting cannot be excluded, and we acknowledge that an assessment of its extent would increase the quality of the data. Another assumption of the Wallinga and Teunis estimation method is that the generation interval remains constant over the course of the epidemic. The sensitivity analysis showed that the shape of the generation interval chosen was important only in the early stages of the outbreak, when a high number of initial cases in the epidemic would equally involve short- and long-distance transmission mechanisms. Another limitation is that in the absence of identified distinct cases resulting from initial virus emergence or introduction, we considered that all cases for the entire epidemic as resulting from transmission from a single index case. In that setting, the initial values of *R_e_* could have been overestimated. If multiple index cases could be identified, the model could be improved by studying transmission within clusters. The algorithm could also be extended to include other information, such as contact between farms caused by movement of animals or environmental data at a higher resolution.

The second limitation is that the 2011 South African RVF dataset was used to build the serial interval distribution because no data from another country or from another epidemic period were available. Although the use of an external dataset would have been more appropriate, the fact that the 2010 and 2011 waves occurred 1 year apart and had a different spatial extent (Figure 1) suggested that both datasets were reasonably independent. However, as a consequence of the 2010 wave, it is possible that vaccination was implemented in the 2011 affected area before the second wave actually started. If applied, vaccination would not modify the shape of the space–time interaction in 2010 and 2011, but it might explain the difference in the intensity of the interactions (*19*). In all cases, the values measuring the intensity of the space–time interaction [*D_0_*(*s,t*)] in 2010 would lie between the 2011 values smoothed with a bandwidth between 1 and 3, and because the sensitivity analysis suggested that the key variations of *R_e_* over time were not affected by these various surfaces, results remain robust.

Another limitation is that vaccine, rainfall, and temperature data used to discuss the plausibility of different reasons for fade-out of the 2010 epidemic were centered on Free State Province. This is where the epidemic started, where vaccination by the government was first applied (*13*), and where 53.9% of the cases were reported by the end of May. Rainfall and temperature data were recorded for Free State Province, which is centrally situated with respect to the outbreak. There is great spatial variation in temperature and rainfall across Free State Province and the country. However, rainfall countrywide was higher than usual that year (*11*,*12*); observations from the field confirmed a decreased winter temperature in Free State Province, starting in April–May (*14*); and trends in environmental variables in Northern Cape Province were similar to those in Free State (Figure 5). 

Furthermore, limited vaccination data were available, so vaccination coverage was estimated under 3 scenarios. It is likely that a large proportion of the 3.4 million vaccine doses sold during April 2009–March 2010 were used in Free State Province at the early stages of the 2010 epidemic (*13*). However, some of those doses would have been used by farmers earlier in 2009 in KwaZulu–Natal and Northern Cape Provinces, where RVF cases were reported and vaccination was applied (*29*,*30*), and in early 2010 because of the perceived risk of further outbreaks. However, detailed figures on this were not available. Therefore, the most likely scenario might have been between Scenarios B and C, corresponding to vaccination coverage of 28.2%–45.7% in Free State Province.

In conclusion, the results of this study suggest that a depletion of RVF-susceptible animals by natural infection or vaccination first contributed to reduce RVF virus transmission in Free State Province and that the effect of further vaccination and the decrease in temperature from April onwards probably helped maintain *R_e_* below unity. Disentangling and quantifying the relative importance of these factors would have benefited from detailed data on monthly vaccine sales and geographic use information. Increasing the public availability of vaccine use data would enable further evaluation of the effect of RVF vaccination campaigns.

Technical Appendix Use of the Wallinga and Teunis algorithm to estimate the effective reproduction number, extended to account for spatial information; formulas used to calculate vaccination coverage; and figure showing distribution of *D*_0_, by time and distance, as a measure of spatiotemporal interaction between cases.
