# Supplementary material for: Transmission Potential of Rift Valley Fever Virus over the Course of the 2010 Epidemic in South Africa
Source: Emerg Infect Dis. 2013 Jun;19(6):916–24. doi: 10.3201/eid1906.121641 (PMC3713830; doi:10.3201/eid1906.121641)
Supplement: Technical Appendix — Use of the Wallinga and Teunis algorithm to estimate the effective reproduction number, extended to account for spatial information; formulas used to calculate vaccination coverage; and figure showing distribution of D0, by time and distance, as a measure of spatiotemporal interaction between cases. [file 12-1641-Techapp-s1.pdf]

Article DOI: http://dx.doi.org/10.3201/eid1906.121641

Transmission Potential of Rift Valley Fever Virus over the Course of the 2010 Epidemic in South Africa

Technical Appendix

Estimation of the effective reproduction number (R_e) by using the Wallinga and Teunis algorithm extended to account for spatial information, and formulas used to calculate vaccination coverage

Estimation of R_e

The Wallinga and Teunis method (*1*) estimates R_e by calculating the relative likelihood that a case *i* gets infected from another case *j* given their difference in time, *t*, of symptoms onset t_i–t_j, and assuming a probability density function (*pdf*) of the generation interval. The generation (or serial) interval is defined as the time between onset of symptoms for a primary case and the onset of symptoms of its secondary case (*2*). The relative likelihood p_{ij} that a case *j* infects a case *i*, is defined as the probability that case *j* infects case *i*, divided by the likelihood that case *i* had been infected from any other case *k* (*1*):

$p_{ij} = pdf \;\; t_i - t_j \;\; / \;\; _{i \neq k} pdf(t_i - t_k),$ Equation 1

The effective reproduction number R_j for case *j* is calculated by summing over all cases *i* infected:

$R_j = \;\; _i\, p_{ij},$ Equation 2

The Wallinga and Teunis likelihood estimation procedure was extended to account for spatial distance between affected farms, so that the likelihood that farm *j* infected farm *i* depended on the time difference between onset of symptoms between the two farms *j* and *i*, and also on their separating distances $|d_i–d_j|$.

Since no independent dataset, that is, from another epidemic in another country was available to estimate a generation interval for RVF at the farm level, and in 2 dimensions

(distance and time), the 2011 RVF South African outbreak dataset was used. In a previous analysis, Métras et al. (*3*) estimated the spatiotemporal interaction (or proximity) from the 2011 dataset [denoted $D_0(s,t)$] using the space–time *K*-function (*4*). These $D_0(s,t)$ values were used as a generation interval distribution in the likelihood estimation procedure (Figure).

Vaccination Coverage

Vaccination coverage (VC) was estimated by March 31, 2010 (end of Period 2, noted VC_2) and May 31, 2010 (end of Period 3, noted VC_3). Since no information on spatial vaccine sales was available, VC was estimated under three scenarios A, B, and C, described as follows. Scenario A assumed that vaccination coverage was applied throughout South Africa, proportionally to livestock population. Scenario B assumed that the number of vaccines used in a province over a specific period of time was proportional to the number of cases reported in that province over that same period of time. Scenario C assumed that all vaccines were used in the Free State province only, during Periods 2 and 3; and that no vaccine had been used before the epidemic (Period 1). Therefore, it allowed estimating the maximum coverage for that province, which was the first and most affected one, and also the one in which the government applied vaccination first (*5*). Corresponding notations were: VC_{A2} and VC_{A3}, VC_{B2} and VC_{B3}; and VC_{C2} and VC_{C3}. Formulas used to calculate VC in the Free State and other provinces are detailed in Table 1. Notations, values and data sources are detailed in Table 2.

References

1. Wallinga J, Teunis P. Different epidemic curves for severe acute respiratory syndrome reveal similar impacts of control measures. Am J Epidemiol. 2004;160:509–16. PubMed http://dx.doi.org/10.1093/aje/kwh255

2. Wallinga J, Lipsitch M. How generation intervals shape the relationship between growth rates and reproductive numbers. Proc Biol Sci. 2007;274:599–604. PubMed http://dx.doi.org/10.1098/rspb.2006.3754

3. Métras R, Porphyre T, Pfeiffer DU, Kemp A, Thompson PN, Collins LM, et al. Exploratory space–time analyses of Rift Valley fever in South Africa in 2008–2011. PLoS Negl Trop Dis. 2012 2012;6:e1808. PubMed http://dx.doi.org/10.1371/journal.pntd.0001808

4. Diggle PJ, Chetwynd AG, Haggkvist R, Morris SE. Second-order analysis of space–time clustering. Stat Methods Med Res. 1995;4:124–36. PubMed http://dx.doi.org/10.1177/096228029500400203

5. AllAfrica.com. Rift Valley fever—South Africa (09): multi-province. ProMed; 2010 Apr 7 [cited 2011 Oct 17]. http://www.promedmail.org, archive no. 20100407.1119.

6. Department of Agriculture, Forestry and Fisheries, Republic of South Africa. Statistical information, livestock numbers—1996 to date. 2011 [cited 2012 May 18]. http://www.nda.agric.za/

7. von Teichman BF, Louw I, Engelbrecht A, Heath JA, Smit TK. Onderstepoort Rift Valley fever virus vaccines. In: Proceedings of the 9th annual congress of the Southern African Society for Veterinary Epidemiology and Preventive Medicine; Pretoria, South Africa; 2010 Aug 18–20; p. 25–31.

8. World Organisation for Animal Health. Rift Valley fever. Follow-up report no. 17. Report reference: Free State Bultfontein, OIE ref: 9982, report date: 2010 Nov 29, country: South Africa [cited 2011 Oct 17]. http://web.oie.int/wahis/reports/en_fup_0000009982_20101129_173322.pdf

9. World Organisation for Animal Health. Follow-up report no 3. Ref: 8937, report date: 08/02/2010, country: South Africa. 2010 [cited 2010 Aug 10]. http://web.oie.int/wahis/reports/en_fup_0000008937_20100208_164327.pdf

10. World Organisation for Animal Health. Follow-up report no. 6. Ref: 8397, report date: 27/08/2009, country: South Africa. 2009 [cited 2010 Sept 10]. http://web.oie.int/wahis/reports/en_fup_0000008397_20090827_173721.pdf

Technical Appendix Table 1. Formulas used to calculate vaccination coverage in Free State Province, South Africa, during the 2010 Rift Valley fever epidemic*

Scenario†	Vaccination coverage at end of Period 2 (VC_2)	Vaccination coverage at end of Period 3 (VC_3)
A	$VC_{A2} = \frac{V_{SA2}}{N_{SA}}$	$VC_{A3} = \frac{V_{SA2} + V_{SA3}}{N_{SA}}$
B	$VC_{B2} = \frac{V_{FS2}}{N_{FS}}$<br>With: $V_{FS2} = \frac{AF_{FS1} + AF_{FS2}}{AF_{SA1} + AF_{SA2}} V_{SA2}$	$VC_{B3} = \frac{V_{FS2} + V_{FS3}}{N_{FS}}$<br>With: $V_{FS3} = \frac{AF_{FS3}}{AF_{SA3}} V_{FS3}$
C	$VC_{C2} = \frac{V_{SA2}}{N_{FS}}$	$VC_{C3} = \frac{V_{SA2} + V_{SA3}}{N_{FS}}$

*Two vaccination coverage (VC) periods were calculated under scenarios A, B, and C: Period 2 (VC_2), ending March 31, 2010, and Period 3 (VC_3), ending May 31, 2010. Notations are detailed in Table 2. Scenario B allowed for the calculation of vaccination coverage for every province in South Africa, using corresponding relevant provincial figures.
†Scenario A assumed that vaccination coverage was applied throughout South Africa in proportion to the livestock population; Scenario B assumed that the number of vaccines used in a province over a specific period was proportional to the number of cases reported in that province over that same period; Scenario C assumed that all vaccines were used in Free State Province during Periods 2 (January 19–March 31, 2010) and 3 (April 1–May 31, 2010) and that no vaccine had been used before the epidemic (Period 1, April 1, 2009–January 18, 2010).

Technical Appendix Table 2. Notation, description, value, sources of the data used to calculate vaccination coverage*

Notation	Description	Value	Source
VC_{A2}	Vaccination coverage in Free State by March 31, 2010, for Scenario A	To estimate	-
VC_{A3}	Vaccination coverage in Free State by May 31, 2010, for Scenario A	To estimate	-
VC_{B2}	Vaccination coverage in Free State by March 31, 2010, for Scenario B	To estimate	-
VC_{B3}	Vaccination coverage in Free State by May 31, 2010, for Scenario B	To estimate	-
VC_{C2}	Vaccination coverage in Free State by March 31, 2010, for Scenario C	To estimate	-
VC_{C3}	Vaccination coverage in Free State by May 31, 2010, for Scenario C	To estimate	-
V_{FS2}	No. vaccine doses sold for Free State during Periods 1 and 2: April 1, 2009–March 31, 2010	To estimate	-
V_{FS3}	No. vaccine doses sold for Free State during Period 3: March 31, 2010–May 31, 2010	To estimate	-
N_{SA}	No. sheep, cattle and goats for South Africa for the period April 1, 2009-May 31, 2010	45,162,901	(*6*)
N_{FS}	No. sheep, cattle and goats for Free State for the period April 1, 2009-May 31, 2010	7,437,641	(*6*)
V_{SA2}	No. vaccine doses sold for South Africa during Periods 1 and 2: April 1, 2009–March 31, 2010	3,400,000	(*7*)
V_{SA3}	No. vaccine doses sold for South Africa during Period 3: March 31, 2010 – May 31, 2010	5,800,000	(*7*)
AF_{SA1}	No. affected farms in South Africa during Period 1: April 1, 2009–January 18, 2010	28	(*8*–*10*)
AF_{SA2}	No. affected farms in South Africa during Period 2: January 19, 2010–March 31, 2010	311	(*8*–*10*)
AF_{SA3}	No. affected farms in South Africa during Period 3: March 31, 2010 – May 31, 2010	151	(*8*–*10*)
AF_{FS1}	No. infected farms in Free State during Period 1: April 1, 2009–January 18, 2010	0	(*8*–*10*)
AF_{FS2}	No. infected farms in Free State during Period 2: January 19, 2010–March 31, 2010	208	(*8*–*10*)
AF_{FS3}	No. infected farms in Free State during Period 3: March 31, 2010 – May 31, 2010	41	(*8*–*10*)

*Scenario A assumed that vaccination coverage was applied throughout South Africa in proportion to the livestock population; Scenario B assumed that the number of vaccines used in a province over a specific period was proportional to the number of cases reported in that province over that same period; Scenario C assumed that all vaccines were used in Free State Province during Periods 2 (January 19–March 31, 2010) and 3 (April 1–May 31, 2010) and that no vaccine had been used before the epidemic (Period 1, April 1, 2009–January 18, 2010).

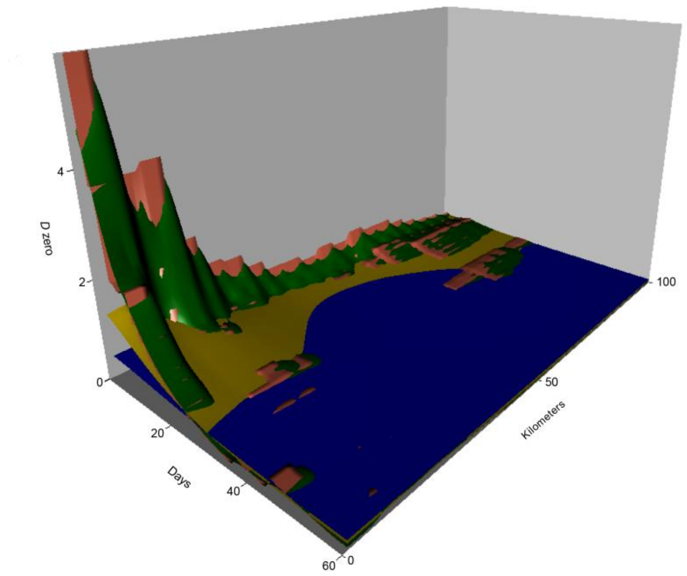

Technical Appendix Figure. Distribution of *D* zero, by time and distance [$D_0(s,t)$]. $D_0(s,t)$ is a measure of spatiotemporal interaction between cases and was estimated by using the space–time *K*-function (*3,4*). Pink surface, $D_0(s,t)$; green, yellow, and blue surfaces are the smoothed distributions obtained with bandwidth values of 1, 3, and 5, respectively.
